# A novel starch-active lytic polysaccharide monooxygenase discovered with bioinformatics screening and its application in textile desizing

**DOI:** 10.1186/s12896-023-00826-1

**Published:** 2024-01-10

**Authors:** Meijuan Zhang, Xiaoping Fu, Rongrong Gu, Bohua Zhao, Xingya Zhao, Hui Song, Hongchen Zheng, Jianyong Xu, Wenqin Bai

**Affiliations:** 1 College of Life Science and Agriculture Forestry, Qiqihar University, Qiqihar, 161006 China; 2 Key Laboratory of Engineering Biology for Low-Carbon Manufacturing, Tianjin Institute of Industrial Biotechnology, Chinese Academy of Sciences, Tianjin, 300308 China; 3 Industrial Enzymes National Engineering Research Center, Tianjin Institute of Industrial Biotechnology, Chinese Academy of Sciences, Tianjin, 300308 China; 4 National Center of Technology Innovation for Synthetic Biology, Tianjin, 300308 China

**Keywords:** Starch-active lytic polysaccharide monooxygenases, LPMO, Hidden Markov model, HMM, Enzymatic textile, Desizing

## Abstract

**Background:**

Lytic polysaccharide monooxygenases (LPMOs) catalyzing the oxidative cleavage of different types of polysaccharides have potential to be used in various industries. However, AA13 family LPMOs which specifically catalyze starch substrates have relatively less members than AA9 and AA10 families to limit their application range. Amylase has been used in enzymatic desizing treatment of cotton fabric for semicentury which urgently need for new assistant enzymes to improve reaction efficiency and reduce cost so as to promote their application in the textile industry.

**Results:**

A total of 380 unannotated new genes which probably encode AA13 family LPMOs were discovered by the Hidden Markov model scanning in this study. Ten of them have been successfully heterologous overexpressed. AlLPMO13 with the highest activity has been purified and determined its optimum pH and temperature as pH 5.0 and 50 °C. It also showed various oxidative activities on different substrates (modified corn starch > amylose > amylopectin > corn starch). The results of enzymatic textile desizing application showed that the best combination of amylase (5 g/L), AlLPMO13 (5 mg/L), and H_2_O_2_ (3 g/L) made the desizing level and the capillary effects increased by 3 grades and more than 20%, respectively, compared with the results treated by only amylase.

**Conclusion:**

The Hidden Markov model constructed basing on 34 AA13 family LPMOs was proved to be a valid bioinformatics tool for discovering novel starch-active LPMOs. The novel enzyme AlLPMO13 has strong development potential in the enzymatic textile industry both concerning on economy and on application effect.

**Supplementary Information:**

The online version contains supplementary material available at 10.1186/s12896-023-00826-1.

## Introduction

Lytic polysaccharide monooxygenases (LPMOs) are copper-dependent redox enzymes secreted by many organisms and viruses [1, 2]. LPMOs catalyze the oxidative cleavage of different types of polysaccharides such as cellulose, chitin, starch and hemicelluloses and have been divided into eight families (AA9-11, AA13-17) within the auxiliary activity enzyme class of the CAZy database [2–4]. Recently, more and more structural and biochemical evidences suggesting that LPMOs follow different mechanistic pathways with different substrates, co-substrates and reductants by behaving as monooxygenases or peroxygenases with molecular oxygen or hydrogen peroxide as a co-substrate, respectively [5]. Thus, the enzymes in different LPMO families would not only be major contributors to the recycling of carbon in nature but also have the potential to be used in various industries such as bio-refinery, biofuels, starch processing industry, and eco-friendly textile, and so on [5].

One research aspect on the substrate range of existing LPMOs showed that excepting the starch-active AA13 family, almost all LPMOs are reported to have activity on β-1, 4-linked polymers of glucose (e.g. cellulose) or N-acetyl-glucosamine (chitin) to date [5]. Starch contains α (1–4) and α (1–6) linkages and exhibits higher order structures compared with cellulose and chitin [6]. The first starch-active LPMO (NCU08746) belong to AA13 family from the fungus *Neurospora crassa* was discovered by bioinformatic analysis in 2014 [6]. The biochemical studies showed that NCU08746 requires copper, oxygen, and a source of electrons to oxidize the C1 position of glycosidic bonds in starch substrates, but not in cellulose or chitin [6]. Whereafter, the oxidative activities of the starch-active LPMOs (NcAA13 and MtAA13) from the fungi *Neurospora crassa* and *Myceliophthora thermophila*, respectively, on three different starch substrates were studied to determine their unique interactions with starch substrates [7]. Thus, the newly found starch-active LPMOs may provide an expanded perspective on application in the food and starch-based bio-processing industries. However, relatively few AA13 family LPMOs have been identified to date, which limits the extended of application range of such starch-active LPMOs.

Based on the increasing number of structural and biochemical characterizations of a growing number of LPMOs, several conserved features were identified. For example, all currently identified LPMOs share a similar core structure dominated by β-sandwich folds and a flat substrate binding surface [8, 9]. Besides, the histidine brace (1st and 2nd conserved histidine) that binds the copper using three nitrogen ligands is fully conserved in all members of all LPMO families [3, 8]. GP Voshol, E Vijgenboom and PJ Punt [8] have developed a novel profile Hidden Markov model (HMM) based on the structure of known LPMOs from the different families (AA9, AA10, AA11 and AA13) and used it to mine genomes of both actinomycetes and ascomycetous fungi for their full content of LPMOs [8]. The correctness of the model has been verified and it indicated that the Hidden Markov model would be an efficient method for novel LPMOs mining. In bioinformatics, the HMM is well-known for its application in modeling the relation between biological sequences in sequence alignment, gene prediction and so on [10]. In sequence alignment, HMM-based methods are used to find relationships and similarity between sequences of DNAs or proteins [10].

Starch, as the second major reservoir of carbohydrates, is not only the major constituent of the human diet but also importance in various industries [11]. Starch and its derivatives account for over 75% of all sizing agents used in the textile industry around the world [11]. Desizing, souring and bleaching are preparatory stages for wet-processing treatments on cotton fabrics [11, 12]. The removal of adhesive compounds (starch and its derivatives) from fabric threads is known as desizing. The adhesive compounds must be applied during the weaving process to prevent yarn from damage and breakage [11, 13]. Amylase has been used in the textile industry to remove starch residues from fabric [11, 13]. To improve the operational efficient and simultaneously reduce its cost, amylases have to be added along with other auxiliary enzymes during the treatment steps [13].

In this feasibility study, we aim to screen a novel auxiliary enzyme of amylase for the potential application in the eco-friendly enzymatic textile desizing. A bioinformatics method which based on the HMM scanning was used to discover new starch-active LPMOs exclusively belonged to AA13 family. The newly found enzyme AlLPMO13 from *Aspergillus lentulus* was successfully expressed in *Escherichia coli* (*E. coli*) and was purified to detect its enzymatic properties. To prove its good application potential in textile industry, the stability in the specific temperatures and pHs for practical application, substrate selectivity, and practical application effect in enzymatic textile desizing have been emphatically determined.

## Materials and methods

### Plasmids, strains and chemicals

The vector pET-32a and *E. coli* trxB (DE3) were used for the heterologous expression of LPMO encoding genes. *E. coli* DH5α was used for cloning of the recombinant plasmids. The commercial mesophilic α-amylase was provided by LONGDA Biotechnology (Shandong, China) Co., Ltd. The amylose, amylose, corn starch, and modified corn starch were purchased from Solarbio Life Sciences (Beijing, China) Co., Ltd. All other chemicals and reagents were purchased through commercial in China.

### Gene mining of the new AA13 family LPMOs by constructing of the Hidden Markov model

Construction of the Hidden Markov model was performed referring to previous reports with minor modified [8, 14]. Firstly, the Hidden Markov model building, database building, and searching rely on the remote server assistance which shared in the bioinformatics platform of our institute. To efficiently access remote servers, the MobaXterm Remote Toolkit was downloaded and installed aforehand on the Windows desktop, which provides all the important remote networking tools such as SSH, FTP, VNC, etc., and Unix commands such as bash, cat, awk, etc. Thereafter, the Anaconda data analysis platform was built, which contains more than 180 science packages and their dependencies, enabling the quick installation of packages frequently used in data science work or the creation of virtual environments to handle multiple projects [15]. The protein sequence files of 34 determined members of AA13 family were searched and downloaded from the NCBI website according to the GenBank accession number marked in the CAZy database. The accession number of each protein sequence in GenBank and the correspondent physicochemical properties of each AA13 LPMO analyzed by Expasy-ProtParam tool (https://web.expasy.org/protparam/) were list in Table S1, respectively. The multiple sequence alignment of the 34 sequences was performed using Jalview (https://www.jalview.org/) software [16] and preserved in stockholm format for the Hidden Markov mode building.

The results of multiple sequence alignment analysis of the 34 AA13 family members were uploaded to the Jackhmmer tool of HMMER online website (http://www.hmmer.org/) for iterative protein search. Iterative search was performed in UniProtKB database with the E-Value of 0.01 and 0.03 for the overall sequence and core motif, respectively, to screen the specific proteins accurately classified to taxonomic distribution of AA13 family. The number of iterative searches was set to 3 times. The taxonomic distribution of AA13 family members in bacteria, archaea, and eukaryotes could be viewed through the results page. At the basis, the appropriate target species would be selected to establish the specific protein sequence database for searching target proteins. Once the target species were identified, the NCBI Datasets tool was used to download the entire proteome file of the target species, and the awk command was used to consolidate the massive sequence data into a single file. After that, the makeblastdb command in the localized BLAST program was used to build the protein sequence database of the target species with the default parameters.

After installing the localized HMMER program [17] via conda environment, the hmmerbuild command was used to train the selected 34 members of AA13 family in the default parameter mode. The HMM dataset reflecting the overall characteristics of the family AA13 can be successfully constructed by comparing and analyzing the results of multiple sequences. Then, using the HMM dataset to scan the target species protein sequence database to obtain the potential members of the family AA13. This process is realized through the hmmsearch command, under which the threshold of relevant parameters can be set to obtain high-quality similar sequences.

### Heterologous expression and purification of LPMOs

The selected LPMO encoding genes were codon optimized and synthesized via GenScript Biotechnology (Nanjing, China) Co., Ltd. The new target genes fused with the 6 × His gene at their C-terminal were cloned in the multiple cloning site (MCS) of the expression plasmid pET32a. Then, the recombinant plasmids were transformed into *E. coli* BL21 trxB (DE3), respectively. The transformants were shake-incubated in terrific broth (TB) medium (100 mg/mL Amp and 50 mg/mL Kan) at 37 °C and 220 rpm [18]. The LPMO expression process induced by isopropyl β-thiogalactopyranoside (IPTG) was performed according to our previous reports [18]. The proteins purified basing on the method reported previously were verified by SDS-PAGE and enzyme assays [19].

### Enzyme activity assay of AA13 family LPMOs by AmplexTM Ultra Red method

The oxygen reactivity of AA13 family LPMOs was measured by a time resolved quantification of H_2_O_2_ formation in 96-well plates [20, 21]. All reactions were performed in 20 mM sodium phosphate buffer, pH 6.0 at 30 °C. 10-acetyl-3, 7-dihydroxyphenoxazine (Amplex Red), horseradish peroxidase (HRP), and ascorbate were used in concentrations of 50 μM, 7.5 U/ml and 100 μM, respectively. In reference experiments without LPMO, the background signal was measured and subtracted from the assays [20]. The LPMO assays were started by mixing 20 μL of sample solution in the reaction system. The fluorescence scans of resorufine were performed using an excitation wave length of 560 nm and an emission wavelength of 590 nm during 30 min reaction. A linear relation between fluorescence and H_2_O_2_ concentrations in the range of 0.1–2 μM H_2_O_2_ was observed and the slope (28,450 counts/μmol) was used for the calculation of an enzyme factor to convert the fluorimeters readout (counts/min) into enzyme activity [20]. The AA13 family LPMO activity was defined as one μmol H_2_O_2_ generated per minute under the defined assay conditions.

### Enzymatic characteristics and substrate selectivity test of LPMOs

The optimum reaction pH of AlLPMO13 was determined at 45 °C in sodium phosphate buffers (20 mM) from pH 4.0 to pH8.0. Stability of AlLPMO13 was measured at different pHs (pH6.0, pH7.0 and pH8.0) and 25 °C for 16 h. The effect of temperature on enzymatic activity of AlLPMO13 was determined at different temperatures from 25 °C to 65 °C in pH 6.0. The thermostability of AlLPMO13 was tested at 25 °C, 40 °C, and 55 °C in pH 6.0 for 16 h, respectively.

Different starchy substrates such as amylose, amylopectin, corn starch, and modified corn starch were used to test the oxidative degradation activity of AlLPMO13 with various dosages (0.01%, 0.03%, and 0.05%, w/v) co-catalyzing with a commercial amylase (with a dosage of 40 U/ml) for 12 h. The reactions without any enzyme addition and with only 0.5% AlLPMO13 and only amylase (40 U/ml) were set as the controls, respectively.

### Enzymatic desizing treatment of cotton fabric by mixed enzymes solution

The enzymatic desizing treatment process of cotton fabric in laboratory was performed according to our previous reports [22]. The fabric test sample was a kind of cotton fabric (20*16, 128*60) sizing with starchy compound slurry. The complex enzymes were prepared by amylase (5 g/L), AlLPMO13 (0 mg/L, 3 mg/L, 5 mg/L and 7 mg/L), H_2_O_2_ (0 g/L and 3 g/L) and penetrant JFC (fatty alcohol-polyoxyethylene ether) (5 g/L). The final dry cotton fabrics were used to test the desizing efficacy using the TEGEWA rating system [11] and measure their capillary effects [22]. The capillary effects of the treated cotton fabric both on the radial and weft directions were determined by the method reported in our previous reports [22].

### Availability of data and materials

The datasets generated and analyzed during the current study are available in the NCBI repository, https://www.ncbi.nlm.nih.gov/guide/. The protein sequences of WD3861, WD3862, WD3863, WD3864, WD3865, WD3866, WD3867, WD3868, WD3869, and WD3870 were respectively corresponded to the accession numbers GAQ05913.1, GFG26764.1, GFF58270.1, KAF7168660.1, PLN83086.1, PYH91397.1, KAF7593475.1, KAE8157273.1, KAH1608114.1, and KAF5860333.1. The DNA sequences with optimized codons which encoding the ten proteins were also deposited in NCBI database (accession No. OR780744-OR7807552).

## Results and discussion

### Screening of new LPMOs belonging to AA13 family through hidden Markov model scanning

**Fig. 1:**
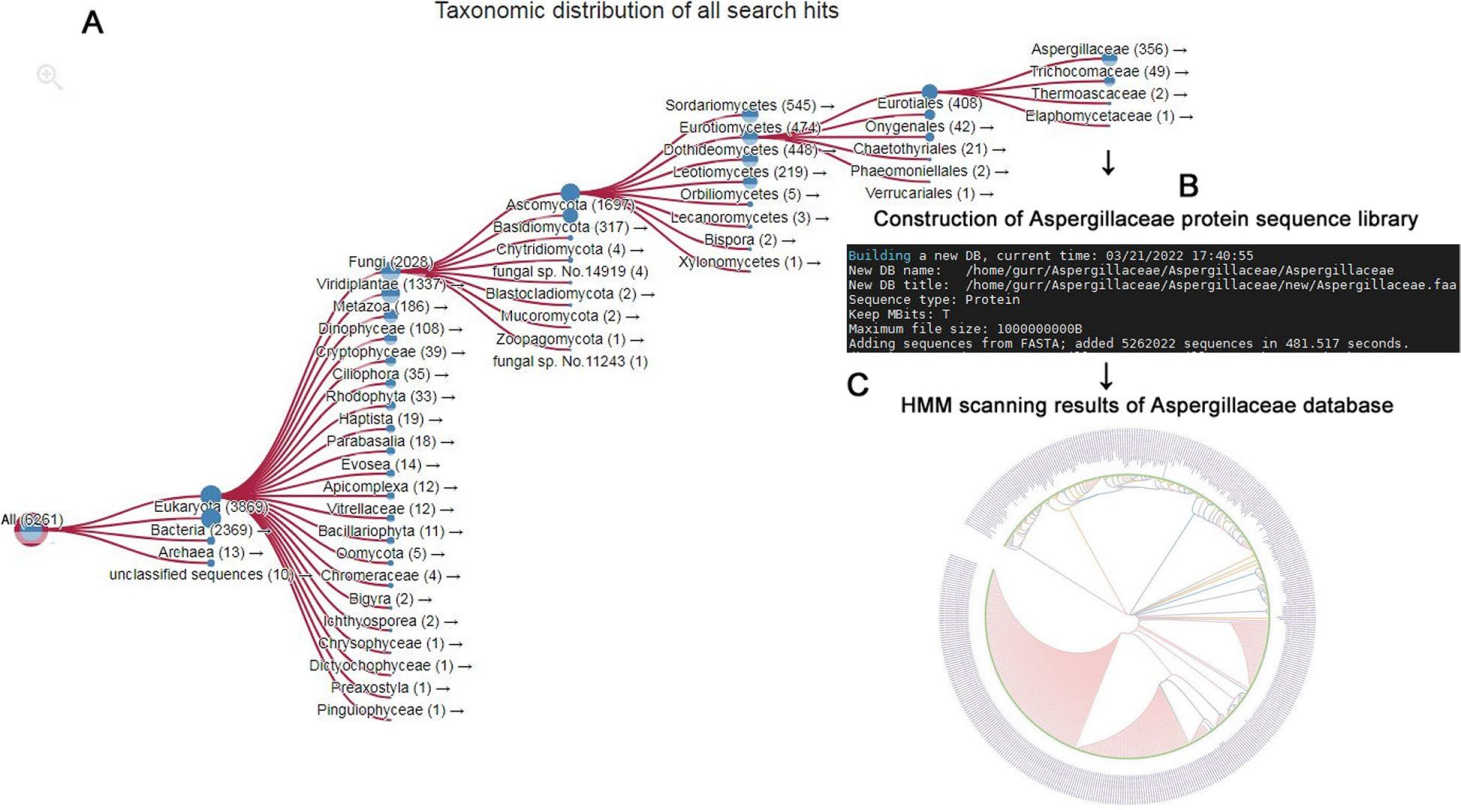
AA13 family LPMO screening process based on the Hidden Markov model. **A** The taxonomic distribution of AA13 family LPMOs after three rounds of iterative searches of the UniProt database. **B** The database construction results of AA13 family LPMO sequences from *Aspergillaceae*. **C** A total of 461 similar sequences with the score at ≥ 450 obtained by hmmsearch

**Fig. 2:**
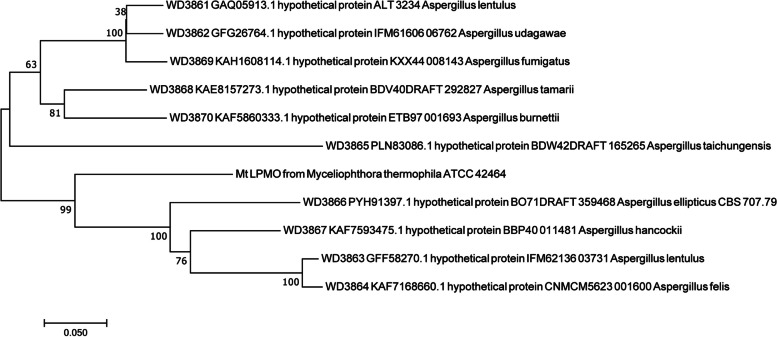
Phylogenetic analysis based on the amino acid sequences of the ten selected AA13 family LPMOs. The reference sequence of MtLPMO was retrieved from GenBank and the phylogenetic tree was built using MEGA 5. Scale bar indicates 0.05 of changes per amino acid position

**Fig. 3:**
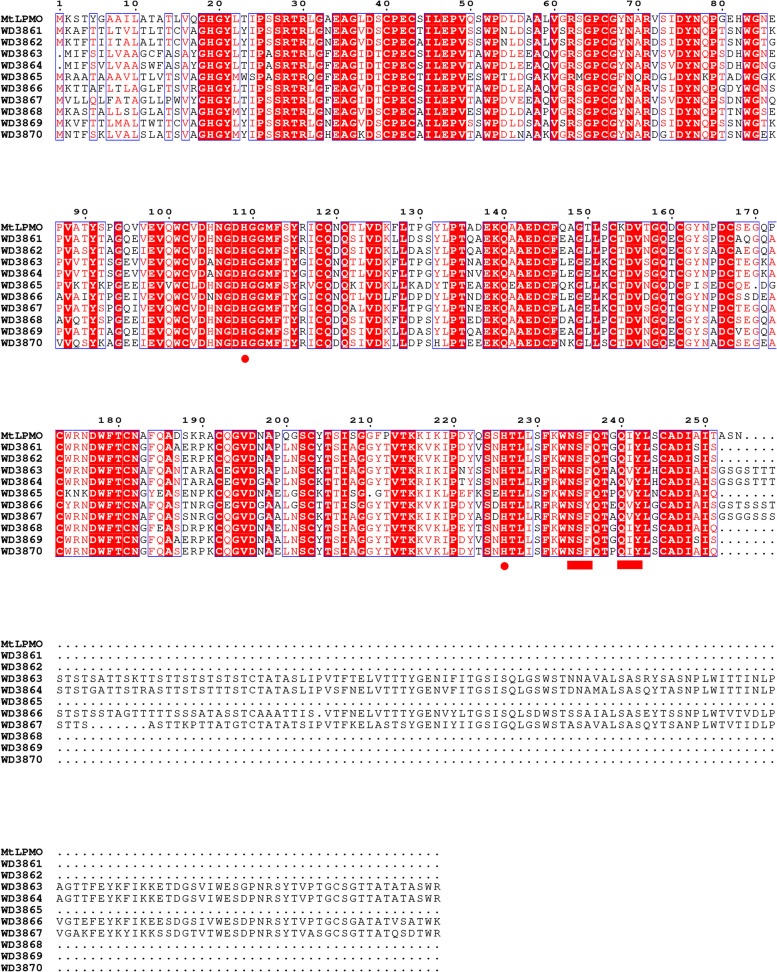
Multiple alignments of the ten selected AA13 family LPMOs and MtLPMO. The conserved amino acid residues were showed with red highlight. The red dots highlighted the conserved histidine residues and the red rectangles highlighted the conserved adjacent N-x-F/Y and Q-x–Y motifs

First of all, multiple sequence alignment analysis of 34 LPMO members of AA13 family which having the specific activity on starch degradation was performed by Jalview (v2.10.5). From the analysis results showed in Figure S1, the 34 proteins showed high similarity in the catalytic domain and the specific sequence features of AA13 family have been identified such as the essential copper binding histidine brace and the N/Q/E-x-F/Y motif [4, 9]. What worth also noticing was that beside the two conserved histidine resides identified before, a new conserved histidine just before the N/Q/E-x-F/Y motif was found in all of the 34 proteins (Figure S1). Moreover, in the 34 LPMOs of AA13 family, there are suspected double N/Q/E-x-F/Y-like motif which showed adjacent N-x-F/Y and Q-x–Y (Figure S1). These sequence features of the 34 starch-active LPMOs would relate tightly with the catalytic and structural properties of AA13 family enzymes. Thereafter, three search iterations of the UniProt database based on the multiple sequence alignment results of the 34 starch-active LPMOs were performed using the Jackhmmer program of the HMMER web server. The taxonomic distribution of AA13 family LPMOs was in sight as shown in Fig. 1A. Based on the analysis results, the members of this family mainly come from eukaryotes and are distributed in about 80 species of fungi in 5 categories of *Ascomycetes* including *Sordariomycetes*, *Eurotiomycetes*, *Dothideomycetes*, *Leotiomycetes*, and *Orbilliomycetes* (Fig. 1A). Among *Eurotiomycetes*, the target sequences were widely distributed in *Aspergillaceae* (Fig. 1A). Thus, a total of 534 proteome files were downloaded from 979 species of *Aspergillaceae* using the NCBI Datasets tool. The AA13 family LPMOs sequence database (AA13PMOdb) from *Aspergillaceae* was built from 534 proteomic files with a total of 5,262,022 amino acid sequences by using the makeblastdb command in localized BLAST program (Fig. 1B). Meanwhile, the Hidden Markov model (HMM13) was built using the hmmbuild program of the HMMER tool version 3 to describe the general sequential features of the AA13 family LPMOs by training the multiple sequence alignment analysis results of all the 34 members of the AA13 family. At the basis, the hmmsearch command was used to scan the AA13PMOdb with HMM13 as the whole sequence score was set as ≥ 450 to mine and obtain highly similar sequences belong to AA13 family. A total of 461 similar sequences were scanned, 380 of which were not clearly described before (Fig. 1C). Ten unannotated new sequences (Table S2) with the highest score values were selected to annotate their protein domains using Batch CD-Search online analysis software. The results showed that all 10 sequences contained absolutely conserved N-terminal AA13_LPMO-like domains and four of them also have the CBM20 domain in their C-terminal (Figure S2). The ten novel enzymatic proteins were named as WD3861, WD3862, WD3863, WD3864, WD3865, WD3866, WD3867, WD3868, WD3869, and WD3870 respectively corresponded to the sequence numbers GAQ05913.1, GFG26764.1, GFF58270.1, KAF7168660.1, PLN83086.1, PYH91397.1, KAF7593475.1, KAE8157273.1, KAH1608114.1, and KAF5860333.1 (Figure S2). The phylogenetic tree and multiple sequence alignment of the ten proteins were built using the protein sequence of MtLPMO from *Thermothelomyces thermophilus* ATCC 42464 as a control, respectively. The ten new enzyme proteins showed various sequence homology to MtLPMO (Fig. 2). WD3866 and WD3861 showed the most and the least homology to MtLPMO, respectively (Fig. 2). It may also lead to diversity in the catalytic activities of the novel AA13-like LPMOs. Compared with the template MtLPMO, the protein sequence similarity of the 10 new enzymes was between 65 and 90%. Through the multiple sequence alignment, the specific sequence features of AA13 family summarized above such as the three conserved histidine residues and the adjacent N-x-F/Y and Q-x–Y were identified (Fig. 3). Thus, the 10 novel enzymes would probably be classified as the starch-activity LPMOs just as we expected.

### Heterologous expression and activity screening of the new LPMOs

**Fig. 4:**
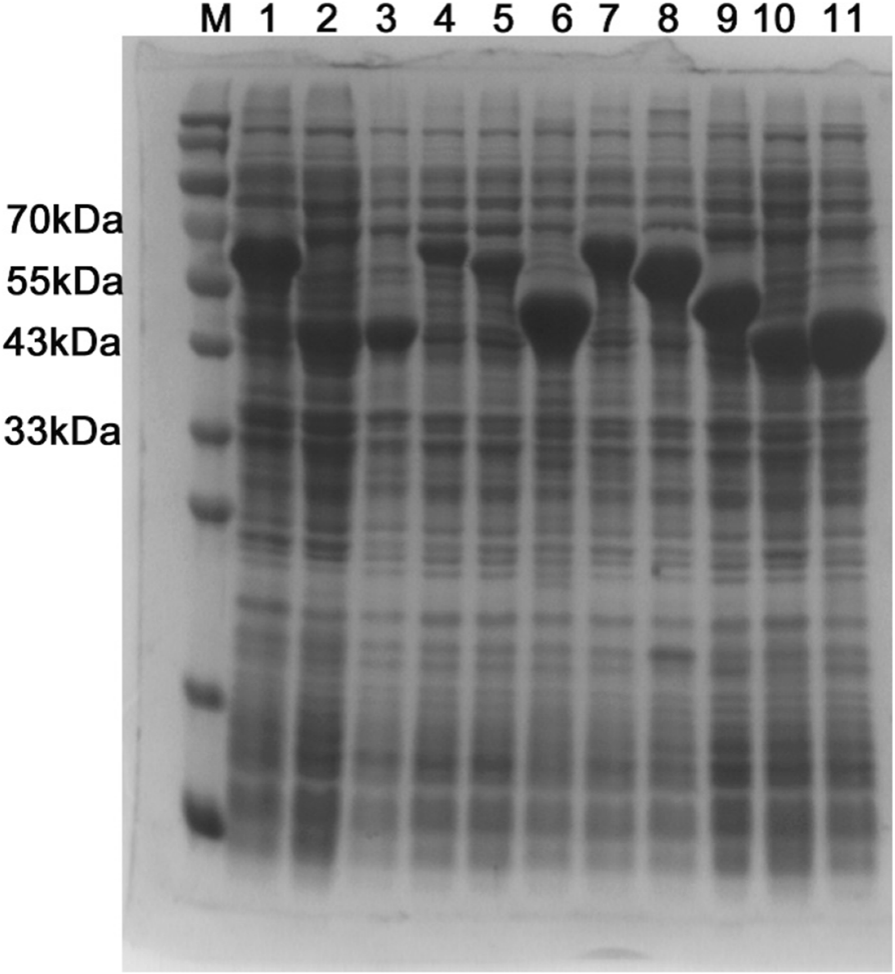
SDS-PAGE of the recombinant LPMOs. lane M: molecular weight of marker standard, lane 1: the positive control, intracellular soluble expression of the recombinant MtLPMO (59 kDa), lane 2–11: intracellular soluble expressions of the recombinant WD3861 (45 kDa), WD3862 (45 kDa), WD3863 (59 kDa), WD3864 (58 kDa), WD3865 (46 kDa), WD3866 (59 kDa), WD3867 (58 kDa), WD3868 (47 kDa), WD3869 (45 kDa), and WD3870 (45 kDa), respectively

**Fig. 5:**
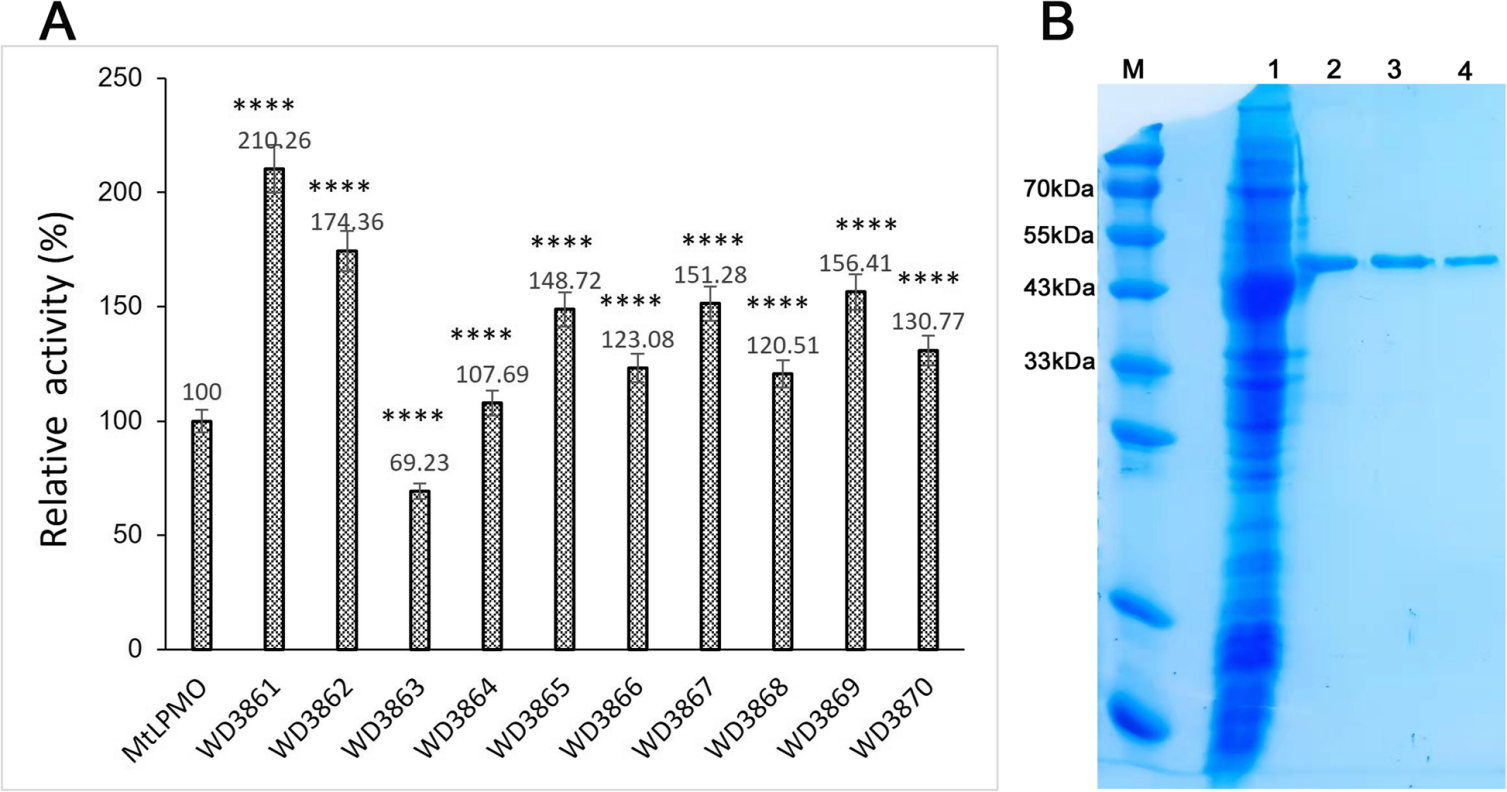
Activity comparation of the recombinant LPMOs (**A**) and purification results of the recombinant AlLPMO13 (**B**). **A** The activity of each enzyme was determined according to the method descripting in the “Enzyme activity assay of AA13 family LPMOs by AmplexTM Ultra Red method” section. The activity of MtLPMO was set as the control (100%). The relative activity of each new screened LPMO was calculated to the percentage against to that of the control. Data of the activities are presented as mean ± SD (*n* = 3). An analysis of variance (ANOVA) and a comparison of the means were conducted with the multiple range comparison LSD test. A probability value of *p* < 0.05 was considered as significant. “****” means *p* < 0.001. **B** lane M: molecular weight of marker standard, lane 1: intracellular soluble proteins of the recombinant strain expressing AlLPMO13, lane 2–4: the purified AlLPMO13 with affinity chromatography

All of the ten new LPMOs have been successfully expressed in *E. coli* BL21 TrxB (DE3) with plasmid pET32a. The results of SDS-PAGE showed that all of the recombinant LPMOs have got intracellular soluble expression after 24 h inducible cultivation with IPTG (Fig. 4). Except WD3862, WD3863, and WD3864, the expression level of the recombinant LPMOs showed comparable levels with that of the recombinant MtLPMO (Fig. 4). However, the enzyme activity test results showed that all of the recombinant LPMOs’ activities were higher than that of the recombinant MtLPMO except WD3863 (Fig. 5). Among them, WD3861 showed the highest activity which was 2.1 times higher than that of MtLPMO (Fig. 5A). Thus, the recombinant WD3861 was further purified to electrophoretic pure protein which showed a single protein band in the test of SDS-PAGE (Fig. 5B). However, as shown in Fig. 5B, the purified protein seemingly showed a relative larger molecular weight than the target protein in the crude enzyme solution. It is probably because that the interaction of proteins with similar molecular weight may cause certain measurement deviation in the SDS-PAGE system. As the sequence of WD3861 was derived from *Aspergillus lentulus* (Fig. 2), the purified protein of the recombinant WD3861 named as AlLPMO13 was used for further researching.

### Characteristics and auxiliary amylase activity of the recombinant AlLPMO13

**Fig. 6:**
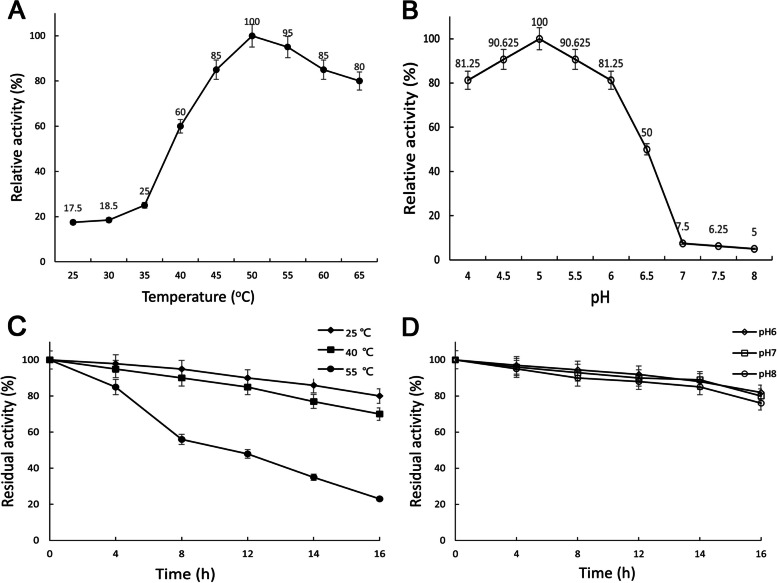
Enzymatic properties of the purified AlLPMO13. **A** The optimum temperature; **(B)** the optimum pH; **(C)** the thermostability; **(D)** the pH stability. The maximal and initial activity of each enzyme was defined as 100%. Data are presented as mean ± SD (*n* = 3)

**Fig. 7:**
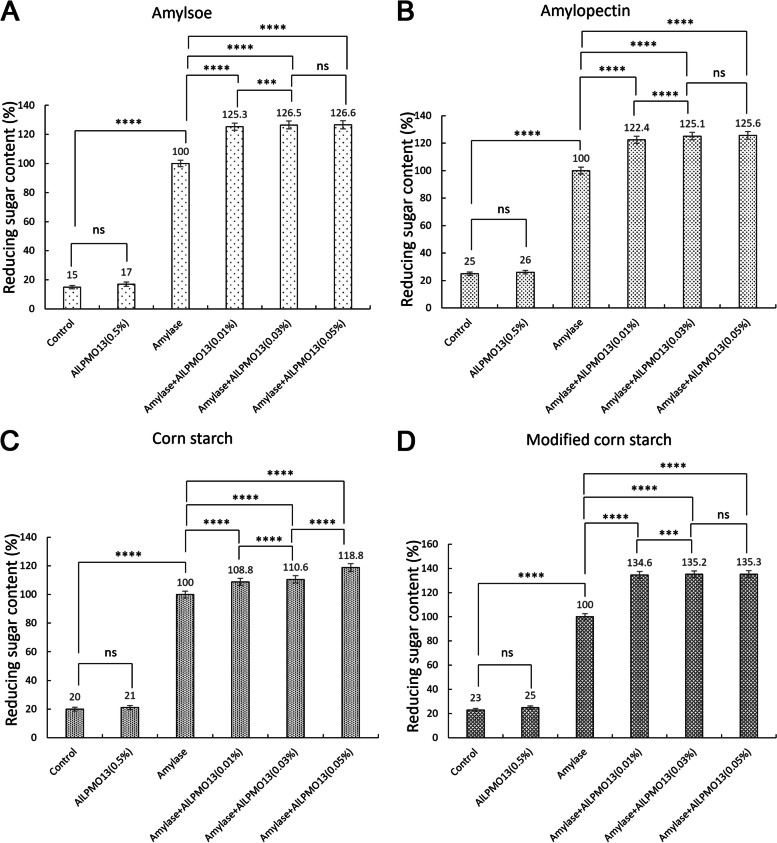
Assisted lytic polysaccharide effect of AlLPMO13 on different substrates. **A** 30% (w/v) amylose as substrate. **B** 30% (w/v) amylopectin as substrate. **C** 30% (w/v) corn starch as substrate. **D** 30% (w/v) modified corn starch as substrate. The reactions of different substrates without any enzyme addition were set as the control groups. The reducing sugar produced by commercial amylase (40 U/ml) catalysis during 12 h was set as 100%. Data are presented as mean ± SD (*n* = 3). An analysis of variance (ANOVA) and a comparison of the means were conducted with the multiple range comparison LSD test. A probability value of *p* < 0.05 was considered as significant. “***” means *p* < 0.005, “****” means *p* < 0.001, and “ns” means *p* > 0.05, respectively

The enzymatic characteristics of the purified AlLPMO13 were detected in different temperatures and pH conditions in this study. As shown in Fig. 6, the optimum reaction temperature and pH were 50 °C and pH5.0, respectively. When at the reaction condition of 45–65 oC and pH4-6, the relative activities of AlLPMO13 were higher than 80% (Fig. 6A, B). These properties make it suitable to be applied with its synergistic enzyme amylase which usually has the maximum enzyme activity at 55 °C and pH6.0 [23]. AlLPMO13 also showed high stability when the temperature was lower than 40 °C and at a pH range of 6–8 during 16 h (Fig. 6C, D). Besides, the amylase-assistant degradation effect of AlLPMO13 on amylose, amylopectin, corn starch, and modified corn starch was assessed to investigate its specificity with various starch polysaccharide substrates. When amylose and modified corn starch were used as substrates respectively, 0.01% additive amount of AlLPMO13 made 25.3% and 34.6% higher reducing sugar product than that been catalyzed alone by amylase (Fig. 7A, D). When the dosage of AlLPMO13 was increased to 0.03% and 0.05%, the reducing sugar increment was very little (Fig. 7A, D). It is probably because amylose and modified corn starch were the optimum substrates of AlLPMO13 that most of their glucosidic bonds could be accessorily broken at a relative lower dosage (0.01%). Thus, when the dosage of AlLPMO13 was increased to 0.03% and 0.05% there was no linear growth trend in the increasement of the reducing sugar. However, when using amylopectin and corn starch as substrates respectively, the reducing sugar increment was 25.1% (with dosages of 0.03% AlLPMO13) and 18.8% (with dosages of 0.05% AlLPMO13) by complex enzyme catalyzing compared to that been catalyzed alone by amylase (Fig. 7B, C). In conclusion, the sequence of the amylase-assistant catalytic activity of AlLPMO13 against different substrates were modified corn starch > amylose > amylopectin > corn starch (Fig. 7). Moreover, according to the results in the Fig. 7, to any substrate (Fig. 7A, B, C, D), the reducing sugar with no remarkably increasing obtained by only AlLPMO13 (0.5%) catalysis compared with the control results which without any enzyme catalysis. It is probably because that the starch polysaccharide chain was cracked by oxidation of AlLPMO13, the order of polysaccharide chain was destroyed, and aldose acid might be produced, or the oligosaccharides released have non-reducing ends, those reaction cannot be detected by DNS. However, the synergistic effect of AlLPMO13 and amylase against different substrates has been indeed determined in this study (Fig. 7).

### The application effect of the recombinant AlLPMO13 on the enzymatic textile desizing

Enzymatic textile desizing mainly relies on the starch degradation activity of amylase for years. However, the composition of the textile sizing becomes more and more complex, the starch slurry is mostly a mixture of different modified starch, which limited the desizing effect of amylase. Some auxiliary enzymes such as pectinase, cellulase and hemicellulase which mainly play the role of refining and can assist to improve the desizing effect. But those auxiliary enzymes are mainly indirect helpers whose real substrate is fiber and not starch. Newly discovered starch-active LPMOs in recent years catalyze the oxidative cleavage of different types of starch substrates could be the optimum auxiliary enzyme of amylase. But so far, no research on textile desizing application of LPMOs has been reported. In this study, since AlLPMO13 showed relative higher degradation activity on modified corn starch which commonly used for textile sizing, it was synergistically used with a commercial amylase on the enzymatic textile desizing application in this study. The dosage of AlLPMO13 was set as around one in a thousand of that of amylase (Table 1). With the increase of AlLPMO13 addition amount, the desizing effect and the capillary effects in both directions of the fabric treated with the complex enzymes were improved accordingly (Table 1). When the dosage of AlLPMO13 was 5 mg/L, the desizing grade was up to 7 and the capillary effects were increased by 16.7% (in radial direction) and 16.2% (in weft direction) times higher than that without AlLPMO13 addition (Table 1). This effect has reached a good level in textile industry. While the desizing grade and the capillary effect was not increased remarkably with the addition amount of AlLPMO13 increased to 7 mg/L. However, in the basis of 5 mg/L AlLPMO13 dosage, an additional 3 g/L of hydrogen peroxide adding could raise the desizing level one more grade and obviously improve the capillary effects (Table 1). It indicated that the oxidation effect of AlLPMO13 on the starch substrates could be remarkably enhanced with increasement of oxygen supply by adding hydrogen peroxide. Besides, the results also showed that adding excess hydrogen peroxide still could not help improve the desizing effect at the condition of sufficient AlLPMO13 addition amount (Table 1). In conclusion, the best combination of amylase (5 g/L), AlLPMO13 (5 mg/L), and H_2_O_2_ (3 g/L) could make the desizing level increased by 3 grade and the capillary effects increased by 21.4% and 20% times in radial and weft direction, respectively, compared with the results treated by only amylase (Table 1). The effect has reached an excellent level in textile industry. It indicated that the novel discovered enzyme AlLPMO13 has a good potential in the application in textile industry.

**Table 1:** Enhanced desizing effects of AlLPMO13 in a textile enzymatic desizing process

Complex enzyme components	Amylase (g/L)^a^						
	5	5	5	5	5	5	5
	AlLPMO13(mg/L)						
	0	3	3	5	5	7	7
	H_2_O_2_ (g/L)						
	0	0	3	0	3	0	3
Capillary effect A^b^	12.6	12.7	13.8	14.7	15.3	15.0	15.1
Capillary effect B^c^	13.0	13.55	14.1	15.1	15.6	15.4	15.8
Desizing grade	5	6	6	7	8	8	8

^a^A commercial amylase with the activity of 4,000 U/ml;

^b^The capillary effect in radial direction of textile (cm/30 min);

^c^The capillary effect in weft direction of textile (cm/30 min)

## Conclusion

The Hidden Markov model basing on the conserved sequences and structure properties of 34 AA13 family LPMOs was proved to be a valid bioinformatics tool for discovering novel LPMO sequences exclusively having starch activity. The new enzyme AlLPMO13 from *Aspergillus lentulus* discovered in this study was also been successfully overexpressed in *E. coli* BL21 trxB (DE3). The high heterologous expression level, high activity, and extensive substrate adaptability of the recombinant AlLPMO13 made it a good prospect of industrial application. The results of an application experiment on the enzymatic textile desizing further proved this point. Compared with the results treated by only amylase, the recombinant AlLPMO13 with one in a thousand of the dosage of a commercial amylase synergying the addition of an oxidative donor (3 g/L) H_2_O_2_ made the desizing level and the capillary effects increased by 3 grade and more than 20%, respectively. It indicated that AlLPMO13 has strong development potential in the enzymatic textile industry both concerning on economy and on application effect.

### Supplementary Information

**Additional file 1: Table S1.** Physicochemical properties of 34 members in AA13 family. **Table S2. **Ten sequences with the highest scores obtained by HMM scanning of the Aspergillaceae database.** Figure S1. **Multiple sequence alignment analysis of 34 members in AA13 family. The red box highlighted the three conserved histidines and the double N/Q/E-x-F/Y-like motif.** Figure S2. **Annotations of domains for candidate expression sequences. 

## Data Availability

All data generated or analyzed during this study are included in this published article and its supplementary information files. All further data will be provided by the corresponding author at any time upon request.

## Abbreviations

LPMO: Lytic polysaccharide monooxygenases
AA: Auxiliary activity
HMM: Hidden Markov model
AlLPMO13: AA13 family LPMO from *Aspergillus lentulus*
MtLPMO: LPMO from *Myceliophthora thermophile*
*E. coli*: *Escherichia coli*
DNS: 3, 5-Dinitrosalicylic acid
IPTG: Isopropyl β-thiogalactopyranoside
JFC: Fatty alcohol-polyoxyethylene ether
CO., LTD: Company Limited
