# Supplementary material for: A novel starch-active lytic polysaccharide monooxygenase discovered with bioinformatics screening and its application in textile desizing
Source: BMC Biotechnol. 2024 Jan 10;24:2. doi: 10.1186/s12896-023-00826-1 (PMC10782670; doi:10.1186/s12896-023-00826-1)
Supplement: Supplementary file 1 — Additional file 1: Table S1. Physicochemical properties of 34 members in AA13 family. Table S2. Ten sequences with the highest scores obtained by HMM scanning of the Aspergillaceae database. Figure S1. Multiple sequence alignment analysis of 34 members in AA13 family. The red box highlighted the three conserved histidines and the double N/Q/E-x-F/Y-like motif. Figure S2. Annotations of domains for candidate expression sequences. [file 12896_2023_826_MOESM1_ESM.docx]

**A novel starch-active** **lytic polysaccharide monooxygenase discovered with bioinformatics screening and its application in textile desizing**

Meijuan Zhang^1,a^, Xiaoping Fu^2,3,4,a^, Rongrong Gu^3,a^, Bohua Zhao^2,3,4^, Xingya Zhao^3,4^, Hui Song^3,4^, Hongchen Zheng^2,3,4,*^, Jianyong Xu^3,4,*^, Wenqin Bai^2,3,4,*^

^1^College of Life Science and Agriculture Forestry, Qiqihar University, Qiqihar, 161006, China.

^2^Key Laboratory of Engineering Biology for Low-carbon Manufacturing, Tianjin Institute of Industrial Biotechnology, Chinese Academy of Sciences, Tianjin 300308, China

^3^Industrial Enzymes National Engineering Research Center, Tianjin Institute of Industrial Biotechnology, Chinese Academy of Sciences, Tianjin 300308, China

^4^National Center of Technology Innovation for Synthetic Biology, Tianjin 300308, China

^a^These authors contributed equally to this work and share first authorship.

^*^Corresponding author. No. 32 West 7th Avenue, Tianjin Airport Economic Area, Tianjin 300308, China.

***Email address:*** [zheng_hc@tib.cas.cn (Hongchen Zheng)](mailto:zheng_hc@tib.cas.cn (Hongchen Zheng)); [baiwq@tib.cas.cn](mailto:baiwq@tib.cas.cn) (Wenqin Bai); [xu_jy@tib.cas.cn](mailto:xu_jy@tib.cas.cn) (Jianyong Xu)

Table S1 Physicochemical properties of 34 members in AA13 family

| Accession number | Numbers of amino acid residues | Molecular weight（kDa） | Theoretical isoelectric point | Total mean hydrophilicity |
| --- | --- | --- | --- | --- |
| VBB78622.1 | 426 | 45.86 | 6.23 | -0.336 |
| QRD84087.1 | 251 | 27.40 | 4.38 | -0.388 |
| QRD05983.1 | 389 | 41.26 | 7.79 | -0.280 |
| QMW44617.1 | 251 | 27.40 | 4.38 | -0.388 |
| QMW32587.1 | 251 | 27.40 | 4.38 | -0.388 |
| QKX59876.1 | 398 | 42.16 | 4.30 | -0.191 |
| QKX53564.1 | 250 | 27.04 | 4.27 | -0.271 |
| QKD48717.1 | 253 | 27.53 | 4.51 | -0.382 |
| QGI98552.1 | 256 | 27.83 | 4.48 | -0.361 |
| QGI84897.1 | 256 | 27.85 | 4.48 | -0.355 |
| QGI267668.1 | 256 | 27.83 | 4.48 | -0.361 |
| QBZ65491.1 | 378 | 40.18 | 5.94 | -0.257 |
| EGX44447.1 | 390 | 41.66 | 6.21 | -0.159 |
| EAU32670.1 | 400 | 42.73 | 4.75 | -0.207 |
| EAQ71571.1 | 353 | 37.22 | 5.24 | -0.266 |
| EAA62623.1 | 385 | 40.79 | 4.68 | -0.139 |
| EAA58078.1 | 328 | 36.35 | 4.73 | -0.386 |
| EAA34371.2 | 385 | 41.00 | 5.17 | -0.130 |
| CZS83188.1 | 253 | 27.67 | 4.48 | -0.396 |
| CEI63409.1 | 253 | 27.61 | 4.58 | -0.409 |
| CEF86045.1 | 253 | 27.62 | 4.42 | -0.384 |
| CDP27694.1 | 426 | 45.85 | 6.24 | -0.339 |
| CCT72014.1 | 253 | 27.52 | 4.48 | -0.374 |
| CCD56877.2 | 403 | 42.20 | 5.17 | -0.099 |
| CBX99030.1 | 404 | 42.38 | 8.07 | -0.259 |
| CAP92263.1 | 435 | 46.32 | 4.88 | -0.236 |
| CAP86689.1 | 248 | 26.85 | 4.50 | -0.273 |
| CAE75704.1 | 405 | 43.29 | 6.31 | -0.154 |
| CAE7027613.1 | 393 | 41.39 | 7.36 | -0.220 |
| BCS19728.1 | 393 | 42.36 | 4.23 | -0.448 |
| BAE61982.1 | 279 | 30.85 | 4.51 | -0.361 |
| ATZ51061.1 | 403 | 42.20 | 5.17 | -0.099 |
| APA15768.1 | 398 | 41.61 | 5.18 | -0.082 |
| AEO62162.1 | 254 | 27.48 | 4.81 | -0.371 |

Table S2 Ten sequences with the highest scores obtained by HMM scanning of the *Aspergillaceae* database

| ---Full sequence--- | | | ---Best 1 domain--- | | | ---#dom--- | | |
| --- | --- | --- | --- | --- | --- | --- | --- | --- |
| E-value | score | bias | E-value | score | bias | exp | N | sequence |
| 4.1e-206 | 692.4 | 17.4 | 4.5e-206 | 692.3 | 17.4 | 1.0 | 1 | KAF7593475.1 |
| 6.5e-204 | 685.1 | 20.7 | 7.3e-204 | 685.0 | 20.7 | 1.0 | 1 | GFF58270.1 |
| 3.1e-203 | 682.9 | 20.6 | 3.5e-203 | 682.8 | 20.6 | 1.0 | 1 | KAF7168660.1 |
| 1.1e-201 | 677.8 | 19.8 | 1.3e-201 | 677.6 | 19.8 | 1.0 | 1 | PYH91397.1 |
| 3.4e-158 | 534.7 | 10.8 | 3.8e-158 | 534.5 | 10.8 | 1.0 | 1 | KAE8157273.1 |
| 1.2e-155 | 526.3 | 12.7 | 1.3e-155 | 526.1 | 12.7 | 1.0 | 1 | GAQ05913.1 |
| 4.8e-155 | 524.3 | 11.0 | 5.3e-155 | 524.1 | 11.0 | 1.0 | 1 | KAF5860333.1 |
| 2.3e-154 | 522.0 | 12.4 | 2.6e-154 | 521.9 | 12.4 | 1.0 | 1 | GFG26764.1 |
| 1e-153 | 519.9 | 13.6 | 1.1e-153 | 519.8 | 13.6 | 1.0 | 1 | KAH1608114.1 |
| 4e-136 | 462.0 | 9.0 | 4.4e-136 | 461.9 | 9.0 | 1.0 | 1 | PLN83086.1 |


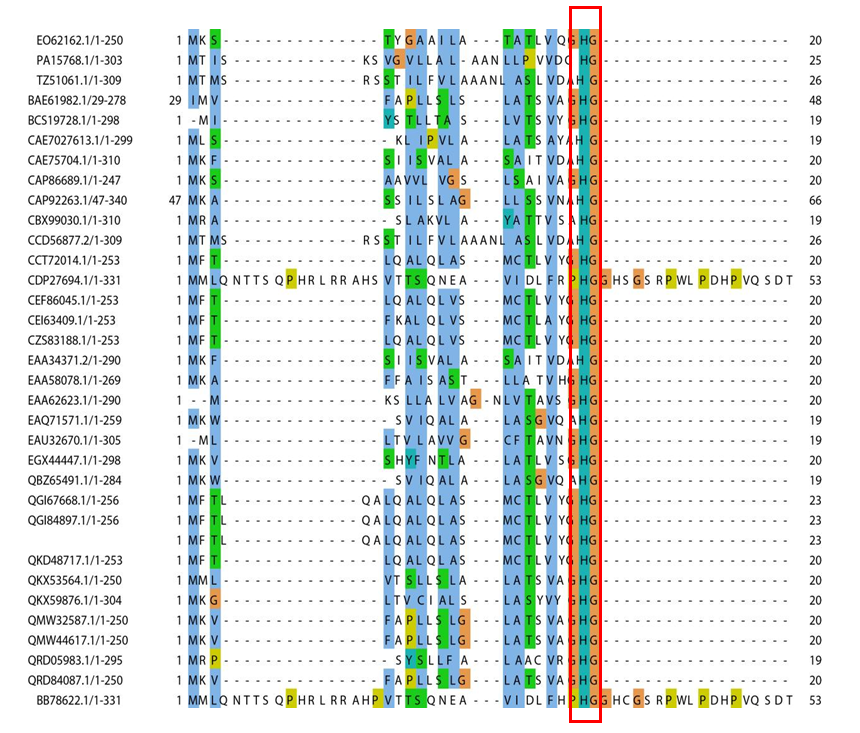


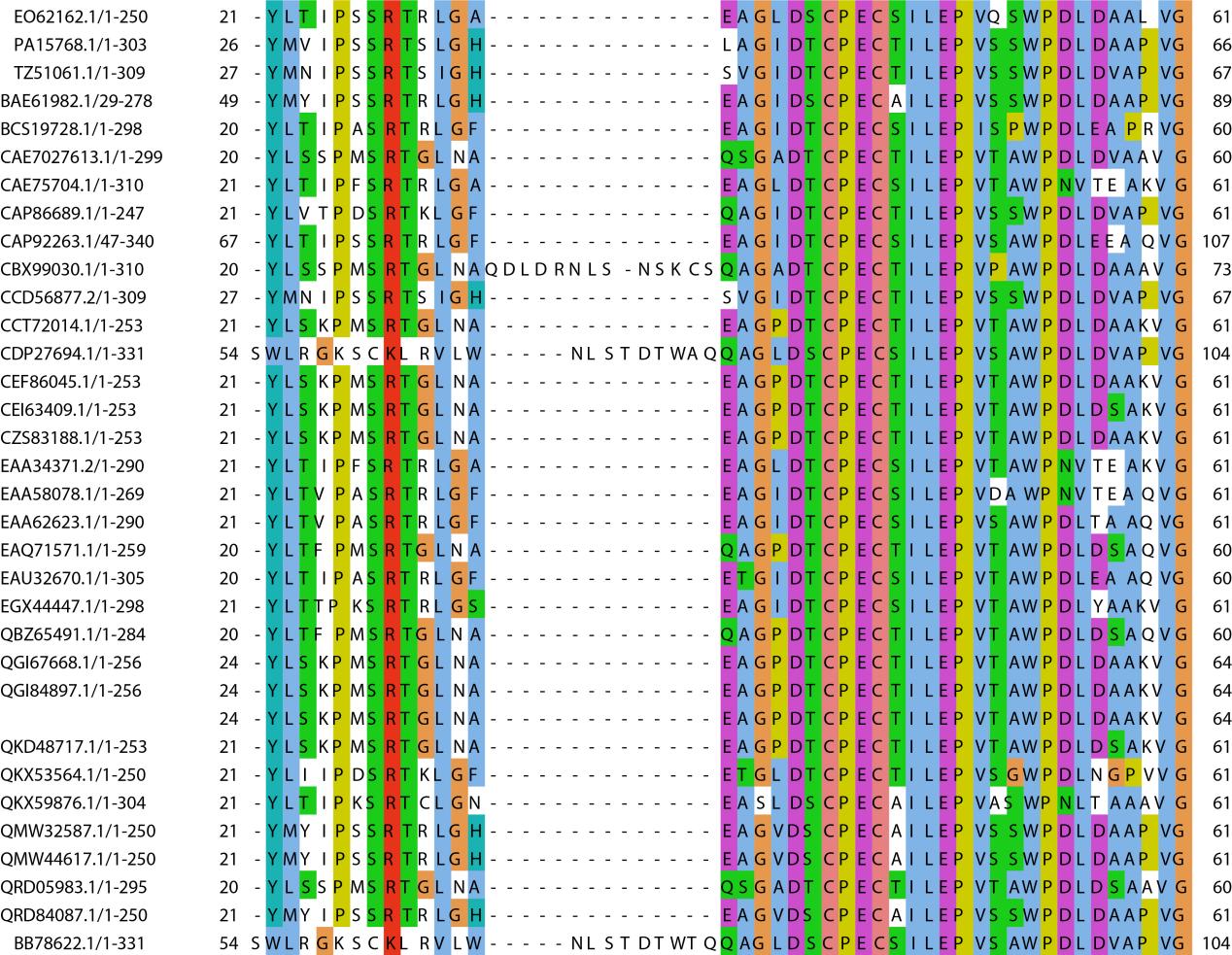


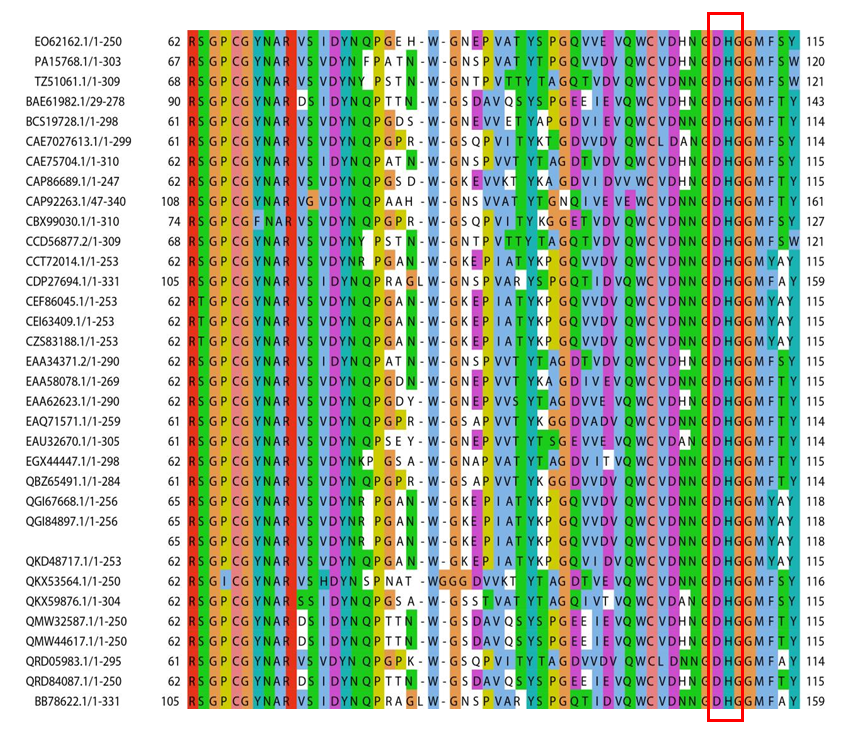


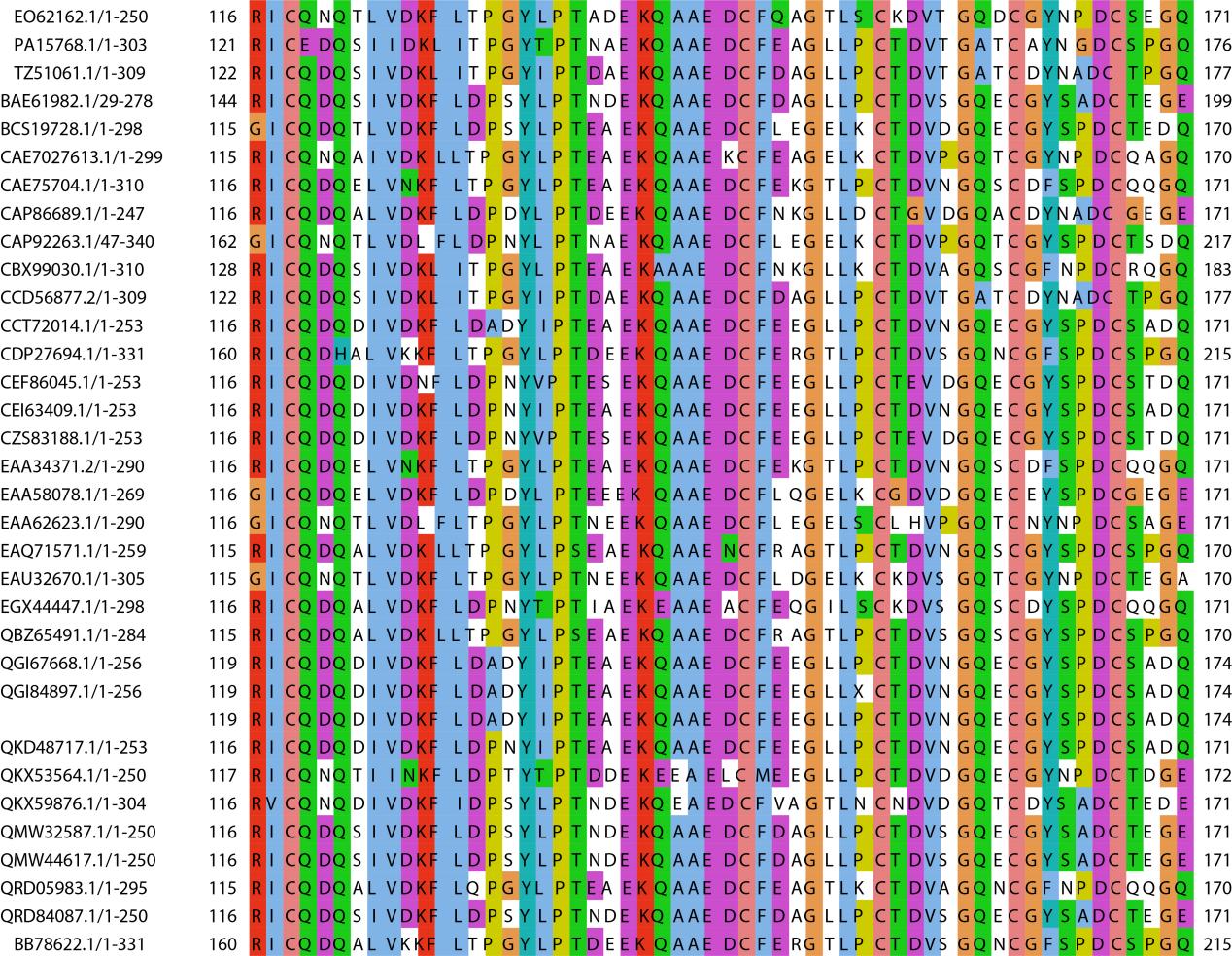

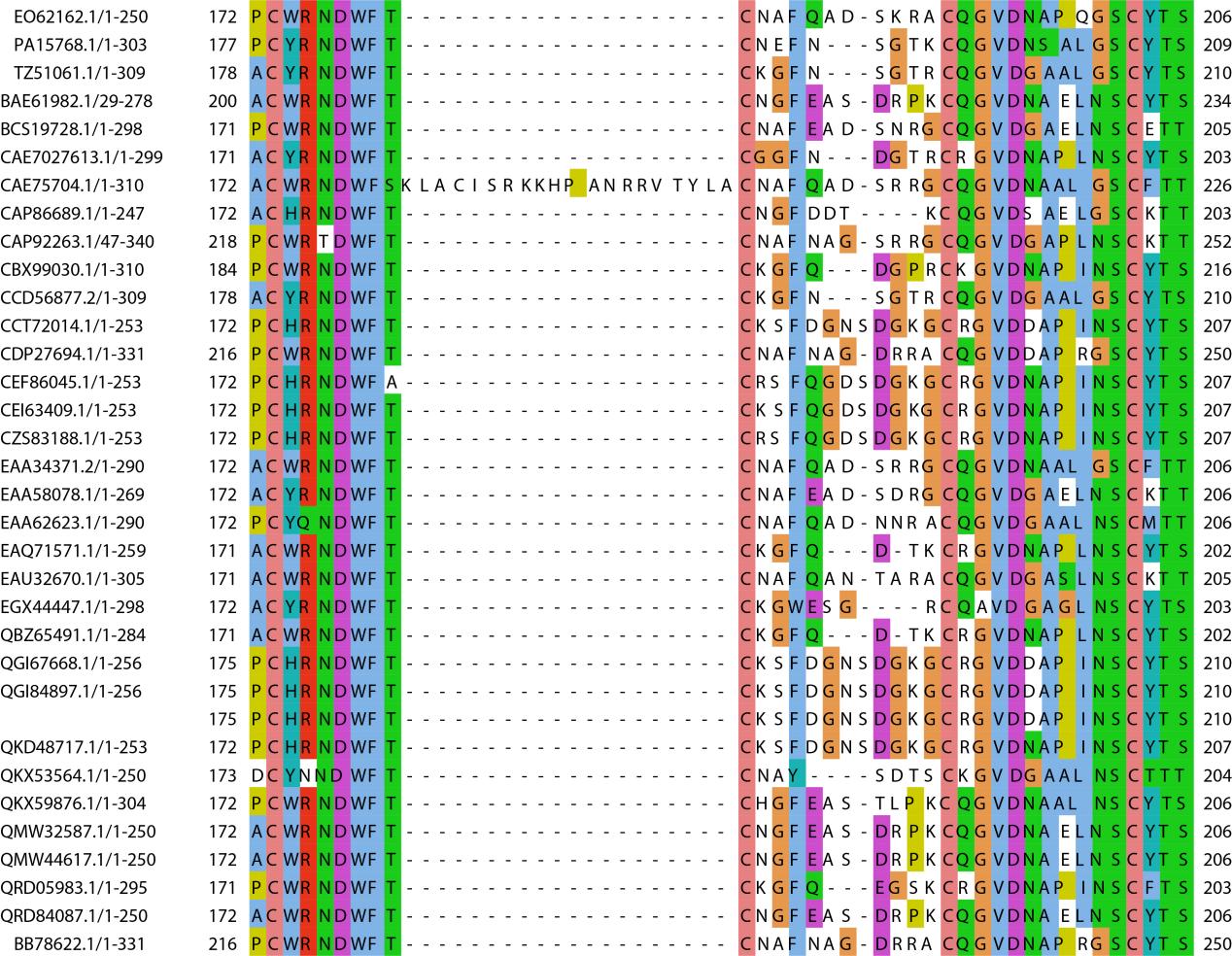


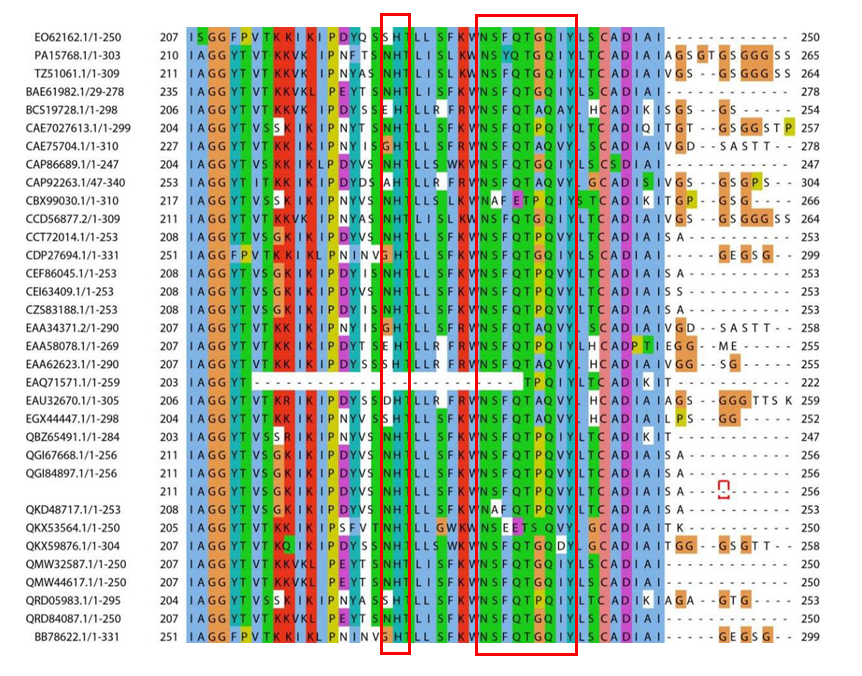

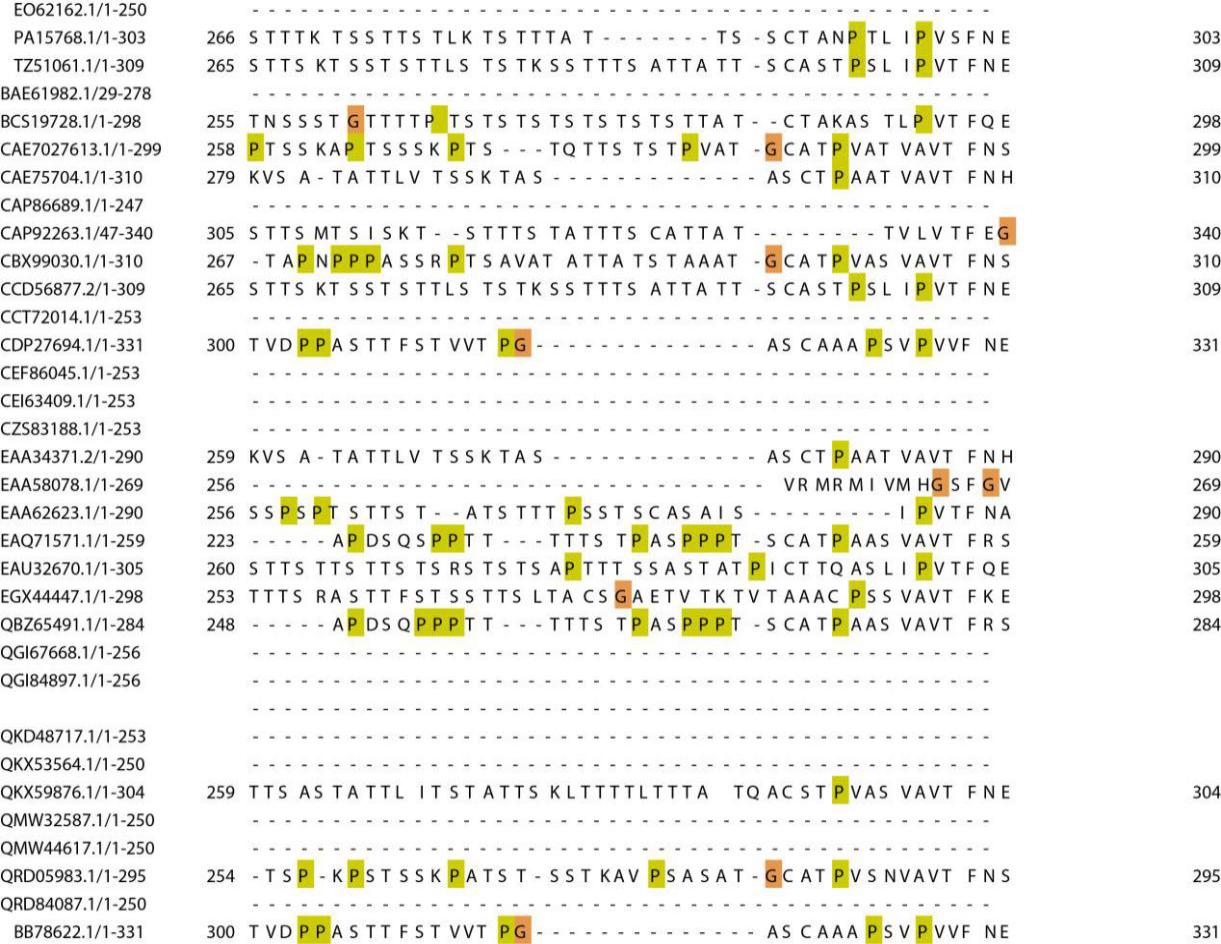


Figure S1 Multiple sequence alignment analysis of 34 members in AA13 family

The red box highlighted the three conserved histidines and the double N/Q/E-x-F/Y-like motif.


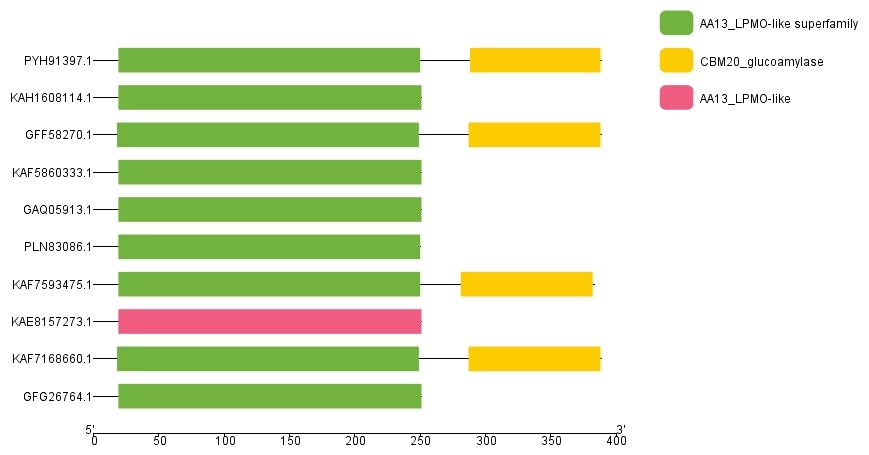


Figure S2 Annotations of domains for candidate expression sequences
